# Integrating Qualitative and Quantitative Data in the Development of Outcome Measures: The Case of the Recovering Quality of Life (ReQoL) Measures in Mental Health Populations

**DOI:** 10.3390/ijerph15071342

**Published:** 2018-06-26

**Authors:** Anju Devianee Keetharuth, Elizabeth Taylor Buck, Catherine Acquadro, Katrin Conway, Janice Connell, Michael Barkham, Jill Carlton, Thomas Ricketts, Rosemary Barber, John Brazier

**Affiliations:** 1 School of Health and Related Research, University of Sheffield, S14DA Sheffield, UK; e.taylor-buck@sheffield.ac.uk (E.T.B.); j.connell@sheffield.ac.uk (J.C.); j.carlton@sheffield.ac.uk (J.C.); t.ricketts@sheffield.ac.uk (T.R.); rosemary.barber@sheffield.ac.uk (R.B.); j.e.brazier@sheffield.ac.uk (J.B.); 2 Mapi Research Trust, 27 Rue de la Villette, 69003 Lyon, France; cacquadro@mapigroup.com (C.A.); kconway@mapigroup.com (K.C.); 3 Centre for Psychological Services Research, Department of Psychology, University of Sheffield, S102TN Sheffield, UK; m.barkham@sheffield.ac.uk

**Keywords:** measuring outcomes, mental health, mixed methods, PROM, quality of life, recovery

## Abstract

While it is important to treat symptoms, there is growing recognition that in order to help people with mental health problems lead meaningful and fulfilling lives, it is crucial to capture the impact of their conditions on wider aspects of their social lives. We constructed two versions of the Recovering Quality of Life (ReQoL) measure—ReQoL-10 and ReQoL-20—for use in routine settings and clinical trials from a larger pool of items by combining qualitative and quantitative evidence covering six domains. Qualitative evidence was gathered through interviews and focus groups with over 76 service users, clinicians, and a translatability assessment. Psychometric evidence generated from data from over 6200 service users was obtained from confirmatory factor models and item response theory analyses. In this paper we present an approach based on a traffic light pictorial format that was developed to present qualitative and quantitative evidence to a group of service users, clinicians, and researchers to help to make the final selection. This work provides a pragmatic yet rigorous approach to combining qualitative and quantitative evidence to ensure that ReQoL is psychometrically robust and has high relevance to service users and clinicians. This approach can be extended to the development of patient reported outcome measures in general.

## 1. Introduction 

With the increasing prevalence of mental health problems [1], there is a need for a short patient reported outcome measure (PROM) to assess the quality of life outcomes for individuals living with mental health conditions. While it is important to treat symptoms, there is a growing recognition of the value of leading a meaningful life and the need to capture the impact of conditions on this, even in the presence of symptoms. It is also known that many people with mental health conditions are able to lead fully “functional lives” despite the presence of symptoms. Most importantly this “recovery” process “is best judged by the person living with the experience” [2] (p. 3). However, it was agreed with the funder of this project that to capture the recovery process, it was important to develop some form of measurement with the following seven criteria. The first and most important criterion was that the measures were based on the outcomes service users identify as being most central to them in recovering their quality of life rather than symptoms [3,4]. The other six criteria were that they should be: available in a version that was short enough for initial assessment and repeated use in routine outcome measurement settings but with a longer version or item set for research purposes; suitable for use with a wide spectrum of mental health conditions and levels of severity; appropriate for individuals aged 16 and over; robust psychometric properties; suitable for self-completion; and free to publicly funded service delivery organisations.

The rationale for developing the new Recovering Quality of Life measures—ReQoL-10 and ReQoL-20—was two-fold. First, existing recovery measures did not meet the above criteria. A systematic review of recovery mental health outcomes assessed 11 instruments for their psychometric properties, ease of administration and service user involvement [5]. None of the measures reviewed met the seven criteria above mainly because they contained too many items, were focused on processes and treatment options which are of course important but not outcomes, they were specific to one patient population or with inadequate psychometric properties (see Supplementary Materials, Table S1). Boardman et al. [3,4] also identified the need for a new measure to contain the themes similar to those suggested by Leamy et al. [6] around connectedness, hope, identity, meaning, and empowerment. Second, in line with the guidelines recommended by the National Institute for Care and Excellence (NICE), EQ-5D is used to calculate benefits to generate quality adjusted life years (QALYs) for use in economic evaluation [7]. However, there is increasing evidence that EQ-5D may not be suitable for some conditions like anxiety [8,9], schizophrenia [10], other psychotic disorders [9,11], and bipolar and personality disorders [12]. Consequently, another preference-based measure may be more desirable for use in the economic evaluation of mental health interventions [13]. Therefore, the ReQoL measures were developed to meet the seven criteria identified above as a routine outcome measure with the possibility of generating a set of preference weights. 

The ReQoL measures were developed in four stages. The theoretical background for the measure which comprised a systematic review of the quality of life (QoL) literature and in-depth interviews with 19 service users identified the following six mental health themes (activity; belonging and relationship; choice and control; hope; self-perception and; well-being) and one physical health theme [8,14,15]. In Stage I, items were generated under each theme using those from existing quality of life and recovery measures; phrases from the interview transcripts used to identify the themes [15]; and items identified by the research team. These items, 1597 in all, were sifted using an adapted criteria list [16,17] to arrive at 87 items. In Stage II, these items were presented in turn to working age adult service users and younger service users to consider their appropriateness. Qualitative data on the items were also gathered on the 61-item set through a translatability assessment (Table 1). In Stage III of the project, psychometric analyses were carried out in two separate studies recruiting 2262 and 4266 participants respectively. The qualitative evidence was integrated with the quantitative data to produce the final measures in Stage IV. In terms of governance of the project, the members of the stakeholders group consisting mainly of policy-makers, representatives from professional bodies, staff from various mental health charities and health care professionals (*n* = 33); the advisory group (*n* = 32) consisting mainly of academics and clinical academics nationally and internationally; and the expert users group (*n* = 6) were asked to comment at each stage of the project. The members of the psychometrics group (*n* = 6) provided specialist advice on the quantitative studies. In addition, the six expert service users were also members of the scientific group (*n* = 18) which formed the decision-making group. 

While it is quite common for both qualitative and quantitative evidence to be used in the development of PROMs, exact details of how the qualitative and quantitative data are combined are often not reported. A possible reason may be because in many cases, the qualitative and quantitative stages are separate stages and the data are used sequentially rather than combining the qualitative and quantitative evidence for the final item selection [18]. The aim of this paper is to present the approach used to combine qualitative and quantitative evidence in the development of the ReQoL measures. While this approach is specific to ReQoL, there is scope for it to be applied more generally to measure development.

## 2. Methods

In Section 2.1 and Section 2.2 below, we summarise the sources, methods, and results of the qualitative and quantitative evidence respectively, followed by the methods used to combine these two types of evidence in Section 2.3. 

### 2.1. Sources, Methods, and Results of Qualitative Evidence 

#### 2.1.1. Qualitative Data from Service Users of Working Age

Fifty-nine service users were recruited from four National Health Service (NHS) Trusts and they were presented with a subset of the 87 items to reduce respondent burden. However, we ensured that service users commented on all the items. This sample is discussed in detail elsewhere [19] in detail, but in summary, the sample included people who had received a range of diagnoses including depression, anxiety, schizophrenia, and personality disorders. The mean age was 42 years and 63% were female. Service users were asked to provide their feedback on the suitability of the items; rephrase items where necessary; and choose their preferred items where there were several covering a similar sub-theme. A pragmatic approach was adopted in the analyses where comments made by each participant were added in a column next to the item in an Excel sheet. Taking into consideration all the comments for each item, a traffic light system was used to highlight items with predominantly negative comments (red), neutral and mixed items (amber), and positive comments (green). 

Items that fell under the following categories were excluded: items not relevant, items that were difficult for service users to respond to; ambiguous items (e.g., items whose meaning was not clear; had more than one meaning), distressing or sensitive items, and judgmental items (e.g., items that imply a certain belief or way of life). Full results of this stage are discussed elsewhere [19].

#### 2.1.2. Qualitative Data from Service Users Aged 16 to 18 Years of Age

As the ReQoL measures would be used in a population as young as 16 years of age, it was essential to ensure that the themes and items resonated with this younger group. The original interviews to identify the themes of the ReQoL measures [15] excluded those aged below 18 and the youngest interviewee was aged 19. Seventeen participants were recruited from two child and adolescent mental health services. All participants were students, and two also worked part-time. They were presented with a set of 61 items but were also asked if any themes were missing. They were consulted on a reduced item set because their interviews happened later in the process and items considered most unsuitable by the adult group were already omitted.

The interviews revealed that while most of the ReQoL themes and items were seen as relevant to the younger service users, there was some indication of a slightly different perspective in relation to some of the themes and items. For example, none of the young people interviewed were living independently; the majority were living with birth parents. They described how living with older adults, who were to some degree responsible for their care, impacted on the degree of choice, control, and autonomy they had. In relation to the self-perception theme, one participant reflected on a degree of confusion about oneself being a normal part of adolescent development. Two final other characteristics of the younger group were a tendency, when talking about their hopes for the future, to focus on academic achievements and motivation and, when discussing the physical health question, there was a tendency to equate physical health with physical fitness and healthy eating. None of the young people had experienced prolonged physical health problems and so the question may have seemed less relevant to them.

#### 2.1.3. Qualitative Data from Clinicians 

Clinicians were consulted throughout the development process but it was deemed necessary to obtain specific feedback at the end of each stage from clinicians who were eventually going to be using the ReQoL measures with their patients. Thirty-five clinicians were interviewed in focus groups from NHS trusts. They were asked to provide feedback on the relevance and usefulness of the items from their viewpoint and whether they already asked the information conveyed by the items as part of their routine consultations [19].

The clinicians involved in the focus groups showed a preference for items that used words, phrases and concepts that they thought were frequently used in conversations with service users. They liked items that were realistic and related to people’s everyday lives, or picked up on issues that they considered important to the people they worked with. The clinicians sought to identify the items that related to functioning and recovery.

Some clinicians stated a preference for items that resonated with things they usually discussed or those that could lead to further conversations. Many clinicians valued the inclusion of questions that could pick up risk and suicidal ideation, as this was something they actively monitored. Clinicians identified certain items from the item set that they did not ask about in their consultation but which they would find helpful as they may indicate the cause and or impact on the mental health components, for example the item “I had problems with my sleep” and the physical health item.

There was debate about whether items should be general or specific, and also about items that required an assessment of other peoples’ thoughts, feelings, or intentions, such as “I felt people did not want to be around me”. Some questions were seen as having the potential to be skewed for particular group of service users. Items that presented a spectrum were preferred to those that presented absolutes such as “Everything in my life felt bad”. Some of the clinicians disliked words that they thought some people might not use or understand and those that had more than one meaning. Whenever there were conflicting views regarding the latter between the clinicians’ and the service users’ views, we adopted the views of the service users. 

#### 2.1.4. Translatability Assessment 

A translatability assessment (TA) was carried out by two linguists (Catherine Acquadro, and Katrin Conway) on the first item set resulting from Stage I (i.e., generation of candidate items—(*n* = 87) (wave 1), and then, on the set of new items generated during Stage II (i.e., content validation—*n* = 11) (wave 2). TA is the review of its source text preferably during the development stage in order to determine its suitability for future translations in multilingual studies. The goal of TA is to facilitate future translations and use of the measure in global studies by: (1) assessing the interpretation of the each item’s underlying concept; (2) identifying and categorising potential translation issues in the source text (either cultural, semantic, idiomatic, syntactic or structural); and (3) providing alternative choices of wordings on which translations can be based and/or recommendations of how to modify the source text (i.e., reformulation or deletion of item) so that future translations are conceptually and culturally appropriate for the target populations [20]. 

In total, on sets of items in both waves (*n* = 98 items), 33 items were classified under a R1 recommendation (no change); 14 under R2 (no change, alternative wording for translation); 19 under R3 (change original), and 37 under R4 (delete item)—see supplementary materials. Five items were put in two classifications (R3 and R4) as the linguists could not decide upon deletion or rewording. Reasons for suggesting deletion were either because of redundancy with other items, inadequacy with response categories, or the strong idiomatic nature of the original. When items were found to duplicate others, the suggestion for deletion was accompanied with a question mark to indicate that a decision had to be made as to which one to delete or keep.

Based on the results of the TA, below are some examples that affected final item selection:

- Rejection of the following idiomatic items “I had reasons to get out of bed in the morning”, “I found it hard to stand up for myself”, “I felt OK about myself”, “I could not bounce back from my problems”
- Rephrasing of the item “I felt at ease with who I am” to “I felt at ease with myself” because of a semantic issue
- Rejection of items with structural issues “I disliked myself”, “I felt unsure about myself” as it was deemed that they might call for translations using a negative verbal form (such as “I did not like…”, “I did not feel sure…”). (See supplementary Tables S2 and S3 for more details).

### 2.2. Quantitative Data 

#### 2.2.1. Study 1 

In Study 1, 2262 service users were recruited from NHS secondary care providers, General practice (GP) surgeries, a trial cohort and voluntary organisations and they were asked to complete a 61-item set. The aim of Study 1 was to reduce the number of items to 40. The mean age of 48 years (range 16 to 97) and participants (58% females) presented with a wide range of conditions including depression, anxiety, schizophrenia, and bipolar and personality disorders. 

#### 2.2.2. Study 2

In Study 2, service users were recruited from similar organisations as in the previous study and they were asked to complete the 40-item set (see Section 3.1 for the results of reducing the number of items from 61 to 40). The mean age was 47 years (range 16 to 98) and services users presented with a wide range of conditions. The samples and results for both studies are described in detail elsewhere [16,21]. 

Factor analyses were carried out in both studies. First, a confirmatory factor analysis (CFA) was undertaken to test whether the six themes adequately represented the structure of the data. Then an exploratory factor analysis (EFA) was carried out followed by a CFA where a two-factor model and a bi-factor model were estimated. Item response theory (IRT) analyses were carried out using graded response models (GRM) to determine the psychometric properties of the items. Classical psychometric analyses were also undertaken, namely responsiveness analyses in Study 2. 

When we analysed the item sets as completed by service users, none of the items had more than 5% missing data and therefore no item was dropped for that reason. The details of the psychometric studies are presented elsewhere [16,21]. In summary, from the factor analysis, 12 highly correlated items highlighting potential redundancy were identified in Study 1 and in Study 2 (Table S4 supplementary materials). From the IRT analyses, in Study 2, four items were identified as misfitting in three of the four datasets. The latter items were: “I felt at ease with who I am”, “I could do the things I wanted to do”, “I had the opportunity to do the things I wanted”, and “I felt safe”. None of the items exhibited any differential item functioning. In Study 2, two items were identified as being insensitive to change “I felt angry” and “I thought people cared about me”. The information functions were generated and scrutinised by theme and the items ranked in order of how much information they provided, whether items were of a middling nature or provided information at extremes. The items providing the poorest information were: “I had the opportunity to do the things I wanted”, “I thought people cared about me”, “I felt angry”, “I felt hopeful about my future”, and “I had problems with my sleep”. 

### 2.3. Combining Qualitative and Quantitative Evidence

#### 2.3.1. Criteria to Summarise Psychometric Evidence 

The first step was to summarise the evidence independently. As explained in Section 2.1.1, the qualitative evidence was colour-coded by one qualitative researcher for the adult service users and another qualitative researcher for the young adult service users and clinicians. The psychometrics group established a set of criteria to choose the items with the best properties (Table 1). 

#### 2.3.2. Combining Evidence in Study 1 

The aim of Study 1 was to further reduce the number of items to around 40 to reduce respondent burden in the subsequent quantitative study. The qualitative and quantitative information were presented in tabular form (see Section 3.1). The main psychometrics evidence used in this stage was highly correlated pairs of items from the factor analysis. The scientific group meeting was chaired by the chief investigator, John Brazier (J.B.). Various researchers synthesised the evidence for each item as follows: psychometrics evidence, Anju Keetharuth (A.K.), qualitative evidence—adult service users, Janice Connell (J.C.), qualitative evidence—younger service users, Elizabeth Taylor Buck (E.T.B.), and translatability assessment (A.K.). Each item was discussed in its theme and the “worse” items were identified for deletion. 

#### 2.3.3. Combining Evidence in Study 2 

With a view to simplifying the information required by members of the scientific group to make a decision for the final item selection, a more intuitive pictorial representation of the evidence was designed. The psychometrics evidence for each item was also colour-coded using the traffic light system by a researcher (A.K.) based on the criteria in Table 1. The various terms used in Table 1 were explained in lay terms to ensure proper understanding. The qualitative evidence was colour-coded by the qualitative researchers (J.C., E.T.B., and T.R. (Thomas Ricketts)). The evidence was then presented in diagrammatic format (see Section 3.2). A similar diagram was prepared for each item under a theme and sub-theme. In cases where two items from different themes were highly correlated as per the factor analyses results and high Spearman correlation > 0.8, this was duplicated under each theme. This document was circulated prior to the scientific group meeting. On the day, a preparatory session was conducted with the expert users group and the clinicians separately to enable members to become familiar with all the materials to enhance their participation in the subsequent meeting. At the scientific group meeting, the items were considered with a view to selecting the best item or items. The criteria for selecting items was to choose those that were qualitatively preferred and with the best psychometric properties. The principles for item selection were as follows:

- Choose one item per theme to retain the face validity of the measure.
- Decide if a second item is needed in this theme; and if so, choose a second item.
- Retain a mix of negatively and positively worded items in the measure.

### 2.4. Ethics 

Ethical approval was obtained from the Edgbaston NRES Committee, West Midlands (14/WM/1062). Informed consent was obtained from all participants in the study. 

## 3. Results 

### 3.1. Study 1 

In Study 1, members of the Scientific Group were presented with this information in advance by theme. An excerpt for the self-perception theme can be found in Table 2. Items were selected from each theme. From the deliberative process, 22 items were deleted at that stage and one item—“I felt miserable”—was added. 

### 3.2. Study 2 

**Figure 1:**
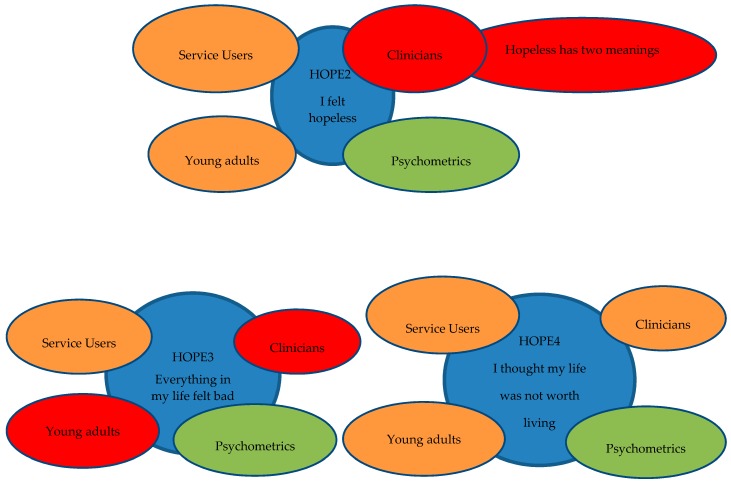
Combining qualitative with quantitative evidence (theme: hope; subtheme: hopelessness). blue: Item; green: Positive views/properties; orange: Mixed views/properties; red: Negative views/properties.

For Study 2, the qualitative and quantitative evidence were colour-coded as illustrated in Figure 1. Diagrams were presented for each theme and sub-theme.

In each theme, the best item based on the colour-coding was first chosen as shown in Table 3. Following a discussion, a decision was reached as to whether a second item under that theme was needed and if so, the second item was chosen using the same procedure. Two themes—self-perception and wellbeing—contain one item only. In the other themes where two items were deemed important, items were chosen such that one was positively worded and one negatively worded. 

In the choice theme, the second item—“I could do the things I wanted to do”—chosen in the scientific committee, had poorer (red) psychometric properties as the item was misfitting in addition to having an inferior information function. The item “I felt hopeful about my future” was also chosen despite having the worst information function in that theme. Both items were discussed at length during the meeting and in the end, and a consensus was reached. In the interest of content and face validity, we chose items that may not have been chosen if only the psychometrics evidence was being taken into consideration.

## 4. Discussion 

This article set out to co-produce a measure of recovery of quality of life by service users and researchers using qualitative as well as quantitative approaches. Broadening the definition of recovery requires an equivalent broadening of the research methods used by combining qualitative and quantitative evidence when developing an outcome measure. We found that it was possible to present the multitude of often complex and technical evidence concisely so that service users, clinicians, and researchers with varying backgrounds in multi-disciplinary teams could equally contribute meaningfully to the final item selection. 

The use of qualitative and quantitative methodologies is the hallmark of a mixed methods approach and has been widely adopted in the research literature [23]. However, it has been less well used in pursuit of measure development. Indeed, virtually all measure development depends solely on quantitative methods with the evidence for validity derived from psychometric analyses. Although we did not follow any formal model for combining qualitative and quantitative methods, our approach has close parallels to the framework advocated by Luyt [24] that, in turn, extended a model initially developed by Adcock and Collier [25]. Luyt’s framework identifies three different levels (theory, domains, and items) that are informed and then refined through an iterative process of combining qualitative and quantitative data. Our informal model broadly followed these levels (termed stages in our approach) with each stage informed by feedback from qualitative analyses (as well as quantitative data), which derived from service users. An interesting component of Luyt’s framework is that the central concept of validity is viewed as being established across methodologies (i.e., both qualitative and quantitative research) rather than multiple aspects of validity (e.g., construct, concurrent, discriminative) being determined within only quantitative methods.

It is obvious that that the qualitative evidence could not have been generated by the researchers alone. The co-production of the ReQoL clearly shows that it is imperative to include service users with lived experience in the development of a measure that is to be relevant to them. The process of excluding service users from the construction of measures is an extension of their exclusion from other activities, and thereby increases their social isolation. By contrast, our view is that service users as well as clinicians, linguists, and researchers all need to be included in the process of production as they all have their own perspectives, life experiences, expertise, and biases. Therefore, co-production is not necessarily the most straightforward way of constructing an outcome measure, but is by far the best way to guarantee a more relevant one. As will have been clear, the ReQoL measures were co-produced in partnership by service users and researchers [26]. However, co-production is only a first step towards social inclusivity whereby service users challenge services to both adopt and implement measures for which service users have a sense of joint ownership. In this sense, the development of measurement tools becomes one other aspect within the area of mental health in which the views of service users need to be central.

While it is paramount to recognise the importance of face and content validity as enhanced by multiple perspectives, it is crucial that an outcome measure assesses what it purports to measure—that is, ensuring that the measure retains the necessary psychometric standards. As shown in Section 3.2, one misfitting item was selected based on qualitative evidence and the deliberation of the members at the Scientific Group meeting. Similarly, an item with a relatively poor information function was also chosen. It is therefore recognised that there has to be a trade-off between superior psychometric properties and the face and content validity. The key in constructing the best possible outcome measure lies in the ability to find the right balance between the two. We think that this has been achieved as the use of the ReQoL is increasing rapidly and the initial psychometric results are encouraging [16]. 

It has been long recognised that qualitative and quantitative can inform each other and be very complementary [27]. However, in health a lack of integration of the two was recognised [28]. The value of this paper is that, instead of keeping the qualitative and the quantitative strands separately, it demonstrates in detail how the two have been successfully integrated. While it is not uncommon, for qualitative work to be carried out alongside psychometrics in measure development, to our knowledge, this is the first paper to use a diagrammatic approach and provide details on the process of integrating the two. The traffic light approach taken by Study 2 is a clear improvement on the tabular depiction in Study 1 and proved easier to understand by all the members of the scientific group. 

There are various ways of using graphics [29] but in this paper we have shown a simple and effective way of presenting complex information to individuals with a view of empowering everyone to participate fully in decision-making. Our evidence shows that it is possible to combine the results from qualitative experts, analysts (psychometricians), clinicians, and service users. However, it is important to be realistic as this approach involves greater planning, time and resources. This way of presenting information can be adopted in many areas of measure development and is reasonably generalisable. 

One caveat regarding the study is that the various groups of adult service users, young service users, clinicians, and linguists assessed different item sets since it was part of an iterative process. For example, at the end of the interviews with the service users, the items that were slightly reworded were not reassessed by the linguists. However, care was taken to ensure that the revised items were in line with the comments received about them. Another caveat is that although the evidence is summarised based on criteria established, the aspect of colour-coding remains subjective. Although the qualitative data transcripts were analysed by three experienced researchers, they were summarised by one researcher only. Similarly, one researcher summarised the quantitative evidence. 

## 5. Conclusions 

The work reported in this article demonstrates that the co-production of the ReQoL outcome measures was enhanced substantially by combining qualitative and quantitative evidence. It is reasonable to suggest that the method of combining qualitative and quantitative evidence discussed in this paper is applicable to outcome measures in general. While it is a valuable process, researchers need to be realistic about the resource implications and be aware of possible trade-offs. We have been able to produce two versions of the ReQoL measure that can be used to assess outcomes of people with mental health conditions, and with future work the ReQoL will also be suitable for calculating quality adjusted life years in the conduct of economic evaluations. 

## Figures and Tables

**Table 1:** Criteria to assess psychometric evidence.

Analyses	Guidance/Judgement/Rule	Determines Exclusion
Missing data at item level	If any item has ≥5% missing data, this item should be dropped Lay description: Respondents do not complete an item maybe because they find it difficult, ambiguous, or simply choose not to answer it.	Yes
Factor analyses	Identify items with high residual correlations (>0.1) Lay description: Assess whether the items measure the different themes of interest and whether the various themes can be aggregated in one score.	Select one item
Item response theory—misfitting items	Identify misfitting items with sum-score based item fit statistic (S-G2) with p values < 0.05 [22] in at least 3 datasets (the sample was randomly distributed in 4 datasets of about 1000 observations in each) Lay description: A mathematical model using all the items is used to explain whether all the items are measuring quality of life (QoL). Items can be identified as misfitting if they are not contributing to measuring QoL. This issue can arise not because of the item but because of the respondents. Therefore, the item can be tested in a different sample before deciding whether to drop it.	No. Acknowledge the misfit but retain item in the item pool.
Item response theory—information functions	Ensure that items cover the whole measurement range (i.e., intensity) by choosing items to balance maximization of information over the total range and content validity (including items from all themes). Lay description: Highly discriminating items provide great information but over a small range of QoL and less discriminating items provide less information but over a wider range of QoL. The graph for each item therefore tells us how much information an item is contributing to the scale and also to what portion of the score range (that those with very low or high QoL).	No
Differential item functioning (DIF)	Exclude items with DIF (age, ethnicity, gender, mental health condition) Lay description: An item is said to display DIF if people with same QoL respond differently to the item because of other characteristic (e.g., by virtue of being female, or belonging to a particular socio-economic group). The item is picking these characteristics up and therefore not correctly representing the true QoL.	Yes
Sensitivity to change	Ensure selected items show change in response over time Lay description: Given that ReQoL would be used routinely over the course of treatment, the items need to be able to register a change in the respondent’s QoL if there is one.	Yes

**Table 2:** Combining the evidence—Study 1 (self-perception theme).

Item	Factor Analyses	Qualitative Evidence			
	Spearman Correlation within Theme > 0.7	Adult Service Users	Younger Service Users	Translatability Assessment	Decision
I felt unsure of myself		Not covered	F (30) A(20) M(50)	✓?	Delete
I tended to blame myself for bad things that have happened	I felt like a failure	F (14) A(8) M(3)	F (80) A(0) M(20)	✓?	Delete
I felt like a failure	I disliked myself	F (15) A(6) M(3)	F (27) A(18) M(55)	✓	Retain
I felt confident in myself	I am at ease with who I am I valued myself as a person I felt ok about myself	F (23) A(3) M(1)	F (64) A(9) M(27)	✓	Retain
I felt at ease with who I am	I valued myself as a person I felt ok about myself	F (15) A(6) M(6)	F (45) A(0) M(55)	✓?	Retain
I valued myself as a person	I felt ok about myself	F (21) A(6) M(1)	F (36) A(0) M(64)	✓	Retain
I disliked myself		F (18) A(6) M(3)	F (45) A(9) M(45)	✓?	Retain
I felt confused about who I am		F (16) A(4) M(6)	F (18) A(27) M(55)	✓? (added about who I am as a result of TA)	Delete
I felt ok about myself		F (19) A(4) M(4)	F (0) A(9) M(91)	“ok”—difficult to translate	Delete

Key: ✓—Fine to select; ✕—Not recommended for selection; ?—mixed; F (for) A (against) M (mixed)—all figures represent percentages; Not covered—this item was not seen by this group; TA—translatability assessment.

**Table 3:** Items chosen under each theme.

Theme	Item Code	Description
Activity	ACT1	I found it difficult to get started with everyday tasks
	ACT2P	I enjoyed what I did *
Belonging and relationships	BEL2	I felt lonely
	BEL3P	I felt able to trust others *
Choice, control, and autonomy	CHO4	I felt unable to cope
	CHO1P	I could do the things I wanted to do *
Hope	HOPE4	I thought my life was not worth living
	HOPE1P	I felt hopeful about my future *
Self-perception	SEL2P	I felt confident in myself
Wellbeing	WB11	I felt happy

* Second item to have been chosen under each theme.

## Supplementary Materials

The following are available online at http://www.mdpi.com/1660-4601/15/7/1342/s1, more details on the translatability assessment; Table S1: Assessing the recovery measures identified by Sklar 2013 by the 7 criteria to be met by a desirable measure Table S2: Results of translatability assessment of first set of items (wave 1 *n* = 87); Table S3: Results of translatability assessment of second set of items (wave 2 *n* = 11); Table S4: Pairs of possible redundant items identified in the factor analyses.

## Abbreviations

The following abbreviations are used in this manuscript:

CFA	Confirmatory Factor Analysis
DIF	Differential Item Functioning
EFA	Exploratory Factor Analysis
GP	General Practice
GRM	Graded Response Model
IRT	Item Response Theory
NHS	National Health Service
PROM	Patient Reported Outcome Measure
QoL	Quality of Life
ReQoL	Recovering Quality of Life
TA	Translatability assessment
