# Supplementary material for: Integrating Qualitative and Quantitative Data in the Development of Outcome Measures: The Case of the Recovering Quality of Life (ReQoL) Measures in Mental Health Populations
Source: Int J Environ Res Public Health. 2018 Jun 26;15(7):1342. doi: 10.3390/ijerph15071342 (PMC6068869; doi:10.3390/ijerph15071342)
Supplement: Supplementary file 1 [file ijerph-15-01342-s001.pdf]

Integrating Qualitative and Quantitative Data in the Development of Outcome Measures: The Case of the Recovering Quality of Life (ReQoL) Measures in Mental Health Populations

Supplementary online materials

Translatability Assessment (TA)

In the TA, an interpretation of the concept for each item has been also provided and constitutes the definition of the items which are currently also being used by translators of the ReQoL measures.

For Stage I, linguists reviewed a set of 87 items distributed in 8 Themes (wave 1): Theme 1—Sense of belonging/relationships, 13 items; Theme 2—Activity: 9 items; Theme 3—Well-being and Ill-being: 24 items; Theme 4—Autonomy/Control/Choice (Empowerment): 9 items; Theme 5—Self Perception: 14 items; Theme 6—Hope/Hopelessness: 8 items; Theme 7—Physical Activity: 7 items, and; Theme 8—Others: 3 items. (In Stage I of the development process, the researchers identified some items that did not fall in any of the seven themes but were deemed important. Therefore, at that stage the others category was temporarily created.) For Stage II, linguists reviewed 11 additional items that were added mainly by service users (wave 2).

The items were analysed, categorised, and recommendations were provided as follows:

- R1: No change to the original wording. The wording of the source text is suitable for international translation and does not require any changes.
- R2: No change to the original wording, but suggestions for alternative wording (based on concept elaboration) suitable for translation to address known issues. This recommendation is suggested when the source wording is the best way to express the concept in the source language but does not translate well.
- R3: Change to the original wording to address issues identified by TA that can threaten the measurement of the concept in other languages.
- R4: Consider removing wording because of extreme degree of difficulty to translate in the future. The developer may decide to keep the item to explore how the item functions during psychometric evaluation before deciding to remove it.

Examples of solutions chosen by the developers are provided below:

- Items that seemed rather idiomatic in a UK and Western context were excluded. They included: *'I had reasons to get out of bed in the morning'* (Theme 2, item 4), *'I found it hard to stand up for myself'* (Theme 4, item 3), *'I felt OK about myself'* (Theme 5, item 2), *'I could not bounce back from my problems'* (Theme 8, item 1). Of note, *'I found it hard to stand up for myself'* was not suggested for deletion by the linguists but for rewording. The item *'I had feelings of* despair' (Theme 3, item 4) was deemed not idiomatic across languages and therefore was replaced by *'I felt despair'*.
- Items with syntactic issues or use of reflexive verbs were also excluded: for instance, '*I neglected myself*' (Theme 2, item 7), although not suggested for deletion by the linguists but to be reworded.
- Having identified a semantic issue in the item '*I felt at ease with who I am*' (Theme 5, item 1), this item was rephrased as '*I felt at ease with myself*' as suggested by the TA linguists.
- Items with potential cultural issue were excluded, for example *'I valued myself as a person'* (Theme 5, item 3). This item explored self-esteem. In some cultures, the concept of valuing oneself as it exists in western countries might not be relevant.
- Structural issues were addressed. '*I disliked myself*' (Theme 5, item 7), *'I felt unsure about myself'* (Theme 5, item 14) were also excluded as it was deemed that they might call for translations using a negative verbal form (such as *'I did not like'*, *'I did not feel sure'*). This would have clashed with the response categories and created double negatives difficult to understand. There were two highly correlated items '*I felt calm*' (Theme 3, item 8) and '*I felt relaxed*' (Theme 3, item 9). In terms of ease of translation 'calm' was preferred, and therefore that item was chosen. Another item changed based on TA was '*I felt hopeful about the future*' (Them 6, item 2) to *'I felt hopeful about my future' on the grounds of the vagueness of* '*the future*'. This problem was also identified by the service users.

In summary, the items that were identified as problematic were, wherever possible, rephrased to facilitate future translation of the measures.

Table S1. Assessing the recovery measures identified by Sklar 2013 by the 7 criteria to be met by a desirable measure.

**Measure**	**Based on CHIME Themes (✓/*X*)**	**(✓/*X*) No of Items**	**Patient Population**	**Age**	**Psychometric Properties**	**Self-complete**	**Free for Use/Acceptability to Users**
Consumer Recovery Outcome System (CROS)	*X*	*X*	? Outpatient	-	(*X*) Based on a single sample – limited validation	✓	*X*
Illness Management and Recovery (IMR) Scale	*X*	✓	?—designed for self-management	-	✓—although not UK based	✓	✓
Maryland Assessment to Recovery in People with SMI (MARS)	*X*	*X*	(*X*) SMI	adults	✓	✓	?
Milestones to Recovery Scale (MTR)	*X*	✓	(*X*) Medium to secure inpatient		*X*	✓	?
Mental Health Recovery Measure (MHRM)	*X*	✓/*X*	(*X*) SMI	adults	✓		
Mental Health Recovery Star (MHRS)					*X*		
Ohio Outcomes System	*X*	*X*	?	adults	*X*	*X*	✓
Peer Outcomes Protocol (POP)	*X*	*X*	*X*	-	*X*	*X*	✓
Recovery Assessment Scale (RAS)	*X*	*X*	*X*	?	✓	✓	?
Recovery Process Inventory (RPI)	*X*—emphasis on processes	*X*			*X*		
Self-Identified Stage of Recovery (SISR)	*X*	✓	✓	adults	*X*	✓	?
Stages of Recovery Instrument (STORI)	✓	*X*	?	adults	*X*	✓	✓
Stages of Recovery Scale	*X*	*X*	?	adults	*X*	✓	✓

Legend: CHIME—the themes identified by Leamy et al (2011) on connectedness, hope, identity, meaning and empowerment; SMI—serious mental illness; ✓—meets the criteria, *X*—does not meet the criteria?—not clear.

Table S2. Results of Translatability Assessment of first set of items (wave 1 n = 87).

**First Set of 87 Items**	**Translatability Issue**						**Recommendations**			
		**Linguistic**				**Structural**	**1**	**2**	**3**	**4**
		C *	S *	P/I *	Sy *					
**Theme 1—Sense of belonging/relationships, 13 items**										
Item 1 I felt loved	✓						✓			
Item 2 I was satisfied with my relationships with others	✓						✓			
Item 3 I felt able to trust others	✓						✓			
Item 4 People close to me caused me distress			✓					✓		
Item 5 I felt humiliated or shamed by other people			✓						✓?	✓?
Item 6 I felt alone and isolated			✓						✓	
Item 7 I had people I could turn to for help				✓		✓—Redundancy with 8				✓
Item 8 I felt nobody cared about me						✓—Redundancy with 7	✓			
Item 9 I felt accepted as who I am	✓						✓			
Item 10 I felt discriminated against			✓			✓—Redundancy with 11				✓?
Item 11 I thought people didn’t want to know me	✓					✓—Redundancy with 10				✓?
Item 12 I felt I made a contribution				✓		✓—Redundancy with 13				✓
Item 13 I was able to do things that helped others	✓						✓			
**Theme 2—Activity: 9 items**										
Item 1 I enjoyed the things I did			✓					✓		
Item 2 I could do all the things I wanted to do			✓					✓		
Item 3 I did things that I found worthwhile			✓					✓		
Item 4 I had reasons to get out of bed in the morning				✓						✓
Item 5 I found the things I did stressful			✓					✓		
Item 6 I felt no interest in things (I found things interesting)			✓			✓—Use of negative formulation that might conflict with RC**			✓	
Item 7 I neglected myself					✓				✓	
Item 8 I could not get started with the simplest everyday tasks						✓—Use of negative formulation that might conflict with RC			✓?	✓?
Item 9 I avoided things I needed to do			✓					✓		
**Theme 3—Well-being and Ill-being: 24 items**										
Item 1 I felt happy			✓			✓—Redundancy with 2				✓?

Item 2 I felt content						✓—Redundancy with 1				✓
Item 3 I had thoughts about killing myself	✓							✓		
Item 4 I had feelings of despair				✓					✓	
Item 5 I felt depressed	✓						✓			
Item 6 I felt miserable			✓					✓		
Item 7 Nothing seemed very much fun (I found things boring)						✓—Use of negative formulation that might conflict with RC				✓
Item 8 I felt calm						✓—Redundancy with 9	✓			
Item 9 I felt relaxed						✓—Redundancy with 8				✓
Item 10 I felt safe	✓						✓			
Item 11 I felt panic or terror			✓						✓	
Item 12 I felt anxious						✓—Redundancy with 14 & 15				✓?
Item 13 I felt scared or frightened			✓						✓	
Item 14 I worried too much			✓			✓—Redundancy with 12 & 15 + inadequacy with RC				✓?
Item 15 I had difficulty stopping or controlling my worrying						✓—Redundancy with 12 & 14				✓?
Item 16 I felt full of life	✓						✓			
Item 17 I felt tired and worn out			✓						✓	
Item 18 I felt everything was an effort			✓	✓		✓—Redundancy with 17				✓?
Item 19 I was thinking clearly	✓						✓			
Item 20 I found it hard to concentrate	✓						✓			
Item 21 I felt irritated	✓						✓			
Item 22 I felt consumed by anger	✓						✓			
Item 23 I have threatened or intimidated another person						✓ - Inadequacy with RC				✓
Item 24 I was disturbed by unwanted thoughts and feelings	✓						✓			
**Theme 4—Autonomy/Control/Choice (Empowerment): 9 items**										
Item 1 I had choices about what I did (I could choose what I did?)			✓			✓—Too vague				✓
Item 2 I had the opportunity to do the things I wanted to do			✓	✓					✓	
Item 3 I found it hard to stand up for myself				✓					✓	
Item 4 I felt trapped	✓						✓			
Item 5 I felt everything was under control				✓		✓—Redundancy with item 8				✓
Item 6 I was able to cope with everyday life	✓						✓			

Item 7 I found ways to deal with life (things?) on a day to day basis			✓			✓—Redundancy with item 6				✓
Item 8 I felt overwhelmed by my problems						✓—Redundancy with item 5				✓
Item 9 I lived as independently as I would like to	✓						✓			
**Theme 5—Self Perception: 14 items**										
Item 1 I felt at ease with who I am			✓			✓—Redundancy with item 2			✓?	✓?
Item 2 I felt OK about myself				✓		✓—Redundancy with item 1				✓?
Item 3 I valued myself as a person		✓	✓							✓
Item 4 I felt confident in myself	✓						✓			
Item 5 I felt useful	✓						✓			
Item 6 I detest myself					✓	✓—Redundancy with item 7			✓?	✓?
Item 7 I disliked myself			✓		✓	✓—Redundancy with item 6				✓?
Item 8 I felt like a failure				✓				✓		
Item 9 I felt useless			✓			✓—Redundancy with item 5				✓
Item 10 I felt stupid	✓						✓			
Item 11 I blamed myself for everything bad that happened			✓			✓—Redundancy with item 13				✓?
Item 12 I felt shame	✓						✓			
Item 13 I felt guilty						✓—Redundancy with item 11				✓?
Item 14 I felt mixed up and unsure of myself			✓			✓—Explores two concepts			✓?	✓?
**Theme 6—Hope/Hopelessness: 8 items**										
Item 1 I thought positively about my future						✓—Redundancy with item 2				✓?
Item 2 I felt hopeful about the future			✓			✓—Redundancy with item 1 + too vague				✓?
Item 3 I had a purpose in life						✓—Inadequacy with RC + Issue of timeframe				✓
Item 4 I felt hopeless						✓— Redundancy with item 4, Theme 3			✓	
Item 5 I didn't think there was anything good in my life (everything in my life feels bad)				✓		✓—Use of negative formulation that might conflict with response categories				✓

Item 6 My life seemed completely pointless						✓—Use of negative formulation that might conflict with response categories			✓	
Item 7 I didn't care about my own life (I thought it would be better if I were dead)						✓—Use of negative formulation that might conflict with response categories			✓	
Item 8 I felt my life was not worth living	✓						✓			
**Theme 7—Physical Activity: 7 items**										
Item 1 I felt physically unwell						✓—Redundancy with item 2				✓?
Item 2 I had physical health problems/difficulties			✓			✓—Redundancy with item 1				✓?
Item 3 I felt fit and healthy			✓						✓	
Item 4 I had problems walking	✓						✓			
Item 5 I had problems with self-care, washing or dressing	✓						✓			
Item 6 I was in pain			✓			✓—Too vague			✓	
Item 7 My health limited physical activities	✓						✓			
**Theme 8—(Others that did not fall in any of the categories above but were deemed important): 3 items**										
Item 1 I felt I could bounce back from my problems				✓						✓
Item 2 I felt confident I could sort out any problems	✓						✓			
Item 3 I had difficulty getting to sleep or staying asleep	✓						✓			

* C: Cultural; S: Semantic; P/I: Pragmatic/Idiomatic; Sy: Syntax; **RC: Response categories.

Table S3. Results of Translatability Assessment of second set of items (wave 2 $n = 11$).

**Second Set of 11 Items**	**Translatability Issue**						**Recommendations**			
	**None**	**Linguistic**				**Structural**	**1**	**2**	**3**	**4**
		C *	S *	P/I *	Sy *					
Item 5 My health limited day to day activities	✓						✓			
Item 28 I felt angry			✓					✓		
Item 29 I had problems with my sleep			✓					✓		
Item 31 I felt terrified			✓					✓		
Item 34 I did things that I found rewarding			✓					✓		
Item 36 I was able to help others	✓						✓			

Item 37 I felt people did not want to be around me				✓				✓		
Item 41 I felt confused	✓						✓			
Item 47 I felt in control of my life	✓						✓			
Item 53 I felt people cared about me	✓						✓			
Item 56 My physical health limited my day to day activities						✓—Possible confusion with item 5				✓

* C: Cultural; S: Semantic; P/I: Pragmatic/Idiomatic; Sy: Syntax.

Table S4. Pairs of possible redundant items identified in the factor analyses

Study 1		Study 2	
**Two-factor model**	**Bi-factor model**	**Two-factor model**	**Bi-factor model**
I felt irritated with I felt angry	I felt irritated with I felt angry	I felt angry with I felt irritated	I felt irritated with I felt angry
I had problems with my sleep with I felt terrified	I had problems with my sleep with I felt terrified	I thought people cared about me with I felt able to trust others	I thought people cared about me with I felt able to trust others
I felt terrified with I felt panic	I felt terrified with I felt panic	I worried too much with I felt anxious	I worried too much with I felt anxious
	I felt like a failure with I tended to blame myself for bad things that have happened	I felt panic with I felt terrified	I felt panic with I felt terrified
	I thought my life was not worth living with My life seemed pointless	I felt anxious with I felt panic	I felt anxious with I felt panic
	I thought nobody cared about me with I felt people did not want to be around me	I felt everything was an effort with I found it difficult to get started with everyday tasks	I felt everything was an effort with I found it difficult to get started with everyday tasks
	I thought nobody cared about me with I thought people cared about me	I felt at ease with who I am with I valued myself as a person	I felt at ease with who I am with I valued myself as a person
	I avoided things I needed to do with I found it difficult to get started with everyday tasks	I could do the things I wanted to do with I had the opportunity to do the things I wanted to do	I could do the things I wanted to do with I had the opportunity to do the things I wanted to do
	I could do the things I wanted to do with I had the opportunity to do the things I wanted to do	I felt people did not want to be around me with I thought people cared about me	I felt people did not want to be around me with I thought people cared about me
	I felt hopeless with I felt lonely	I felt terrified with I felt safe	I felt terrified with I felt safe
	I worried too much with I felt anxious		I avoided things I needed to do with I found it difficult to get started with everyday tasks
	I felt terrified with I felt safe		I felt calm with I felt relaxed
